# Genetic variants in *RTEL1* influencing telomere length are associated with prostate cancer risk

**DOI:** 10.7150/jca.35917

**Published:** 2019-10-15

**Authors:** Cheng-Yuan Gu, Sheng-Ming Jin, Xiao-Jian Qin, Yao Zhu, Dai Bo, Guo-Wen Lin, Guo-Hai Shi, Ding-Wei Ye

**Affiliations:** 1 Department of Urology, Fudan University Shanghai Cancer Center, Shanghai, China;; 2 Department of Oncology, Shanghai Medical College, Fudan University, Shanghai, China.

**Keywords:** prostate cancer, polymorphisms, telomere, susceptibility

## Abstract

Telomere length measured in lymphocytes has been evaluated as a potential biomarker for prostate cancer (PCa) risk. Identifying genetic variants that affect telomere length and testing their association with disease could clarify any causal role. We therefore investigated associations between genetic variants in three telomere length-related genes and PCa risk in a case-control study. The influence of these variants on the leukocyte telomere lengths was then appraised by real-time PCR. *RTEL1* rs2297441 [odds ratio (OR): 1.23; 95% confidence interval (CI): 1.03-1.46, *P* = 0.021] and rs3208008 (OR: 1.23; 95% CI: 1.03-1.46) were associated with PCa risk. These two risk single nucleotide polymorphisms (SNPs) (OR: 0.59; 95% CI: 0.39-0.89, *P* = 0.012 and OR: 0.58; 95% CI: 0.38-0.87, *P* = 0.009, respectively) and another SNP *PARP1* rs1136410 (OR: 1.53; 95% CI: 1.01-2.31, *P* = 0.043) were also associated with leukocyte telomere length. These findings support that genetic determinants of telomere length may influence PCa risk.

## Introduction

Telomeres are TTAGGG nucleotide repeats and a protein complex at chromosome ends that play an essential role in maintaining chromosomal stability. Due to the inability of DNA polymerase to fully extend 3' DNA ends, telomeres become gradually shorter with each cell division in the absence of telomerase activity^1^. Although in normal cells critically short telomeres will trigger cellular senescence and death, cancer cells can maintain the abnormal short telomere length for unlimited growth by telomerase activity reactivation 2, 3. Therefore, telomere length is an important determinant of telomere function. Indeed, recent studies suggest telomere length may be a risk factor for tumor types including prostate cancer (PCa)^4^.

Telomere length is influenced by inherited genetic factors^5^. Genome-wide association studies (GWAS) have shown that telomere length is influenced by inherited genetic polymorphisms containing known telomere-related genes^6^, ^7^. The existence of genetic variants influencing both telomere length and cancer susceptibility explained the association between telomere length and cancer risk. Recent Mendelian randomization approaches have shown that identified significant single nucleotide polymorphisms (SNPs) for leukocyte telomere length could be used as a risk marker for several cancer types^8^, ^9^. Previous studies attempted this for PCa and found several variants in telomere structure and maintenance gene regions were associated with PCa risks, although telomere length were not measured meanwhile^10^.

In the present study, we evaluated six potentially functional genetic variants from three telomere length-related genes (*RTEL1*, *POT1* and *PARP1^1^*) in relation to PCa risk in a case-control study. We also evaluated whether these genetic variants are associated with leukocyte telomere length to investigate a potential etiologic relationship.

## Materials and Methods

### Study Design and Population

The study subjects were mostly from previously published case-control study^11^-^13^. Briefly, 1015 eligible patients recruited into this study were newly diagnosed and histopathologically confirmed primary prostate adenocarcinoma from Fudan University Shanghai Cancer Center (FUSCC) between January 2005 and January 2012. All cases had received no prior chemotherapy or radiotherapy upon recruitment. The tumor stage was determined according to criteria established by the American Joint Committee on Cancer (AJCC) tumor-node-metastasis (TNM) classification system [AJCC Staging Manual, sixth edition, 2002]. Histopathological grading of the specimens was performed according to the Gleason score system. The clinical information including Gleason score, serum PSA level at diagnosis and disease stage were abstracted from the archival medical records. In addition, 1143 age-(± 5yr) and geographical regions-matched cancer-free ethnic Han Chinese controls were recruited from the Taizhou longitudinal (TZL) study conducted during a similar time period. TZL study was a large prospective cohort initiated to explore the environmental and genetic risk factors for common non-communicable diseases. Individuals with a known test of serum PSA > 4 ng/mL present with or without abnormal digital rectal examination were excluded from the control group and those without response to the study participation were excluded (n = 91). All of the participants were interviewed with a questionnaire after a written informed consent was obtained. Blood samples were collected and processed as a routine practice by the FUSCC Tissue Bank (for cases) and the TZL study (for controls). This study was approved by the Institutional Review Board of FUSCC.

### SNP Selection and Genotyping

We searched the National Center for Biotechnology Information dbSNP database (http://www.ncbi.nlm.nih.gov/) for potentially functional SNPs and SNPinfo (http://snpinfo.niehs.nih.gov/) to identify the candidate SNPs based on the following three criteria: (1) located at the regulatory or coding region of genes (i.e., the 5' near gene, 5' untranslated regions [UTR], exons, splice sites, 3' UTR and 3' near gene); (2) the minor allele frequency (MAF) ≥ 5% in Chinese Han, Beijing descendants reported in HapMap; (3) affecting the activities of microRNA binding sites in the 3' UTR and transcription factor binding sites in the putative promoter region or changing the amino acid in the exons. All these six SNPs were genotyped by the TaqMan real-time PCR method as described previously^11^-^14^. Briefly, DNA isolation was performed by using the Qiagen Blood DNA Mini KIT (Qiagen Inc., Valencia, CA) with the buffy-coat fraction of the blood samples donated by the participants. The results with > 99% call rates and 100% concordance for duplicated specimens were acceptable for further genotyping data analysis.

### Measurement of Relative Telomere Length

Telomere length measurements were available in 426 patients who received radical prostatectomy as described previously^12^, ^14^. Briefly, leukocyte telomere length was measured using real-time quantitative PCR method on an Applied Biosystems 7900HT. The PCR reaction mixture consisted of SYBR Green Mastermix (Applied Biosystems), 100 nmol/L Tel-1, 900 nmol/L Tel-2, 400 nmol/L 36B4d, 400 nmol/L 36B4u, and 7ng of genomic DNA. Two main steps were involved in telomere length quantification: first, the T/S ratio was determined for each sample based on the standard curve. Second, the ratio for each sample was normalized to the calibrator DNA to standardize sample values across all reaction plates. The laboratory personnel were blinded to disease status. R^2^ for each standard curve was ≥ 0.99.

### Statistical Analysis

For all subjects, the χ^2^ test was used to assess differences in the frequency distributions of the selected demographic variables and genotypes of six SNPs between the cases and controls. The Hardy- Weinberg equilibrium for genotype distribution in controls was tested by a goodness-of-fit χ^2^ test. Odds ratios (ORs) and 95% confidence intervals (CIs) were calculated by univariable and multivariable unconditional logistic regression models to evaluate associations between the genotypes and risk of PCa without and with adjustment for confounding factors, respectively. Spearman rank correlation was used to investigate associations between telomere length and age. Telomere length was categorized into dichotomies based on the distribution. All statistical analyses were performed with SAS software (version 9.1; SAS Institute, Cary, NC).

## Results

### Characteristics of the study subjects

The distributions of demographic characteristics of the subjects are shown in **Table 1**. Briefly, there were no statistical differences in the distributions of age and smoking status between two groups.

### Genetic variations and PCa Risk

The genotype frequencies of six SNPs and their associations with PCa risk are summarized in **Table 2**. The observed genotype frequencies of six SNPs in controls agreed with the Hardy-Weinberg equilibrium. Furthermore, the genotype distribution of *RTEL1* rs2297441 and rs3208008 was significantly different between the cases and controls. In multivariate logistic regression analysis, *RTEL1* rs2297441 and rs3208008 were associated with PCa risk. Compared with GG genotype, rs2297441 variant AA genotype was associated with an increased risk of PCa (OR: 1.44, 95% CI = 1.08-1.93). Compared with AA genotype, rs3208008 variant AC/CC genotype was associated with an increased risk of PCa (OR: 1.23, 95% CI = 1.03-1.46). However, no associations between the other four SNPs and PCa risk were observed.

### Genetic variations and telomere length

To investigate whether the telomere length had prognostic significance, 426 patients were categorized into dichotomies based on their telomere length distribution (range, 0.06-2.06). Telomere length was inversely associated with age (r = -0.40, *P* < 0.001). When participants were dichotomized according to the median telomere length value, significant differences in telomere length by genotype of *RTEL1* rs2297441, *RTEL1* rs3208008 and *PARP1* rs1136410 were observed (**Table 3**). Compared with GG genotype, rs2297441 variant AA genotype was associated with shorter telomere length (OR: 0.50, 95% CI = 0.39-0.89).

## Discussion

In the present study, we identified that the *RTEL1* rs2297441 and rs3208008 variant genotypes were significantly associated with PCa risk. The association between PCa risk and these two SNPs in the *RTEL1* gene was further supported by the telomere length analysis. Our results indicate that a genetic predisposition to shorter telomere length may be a risk factor for PCa.

*RTEL1* encodes an essential iron-sulfur (FeS)-containing DNA helicase that is critical for facilitating telomere replication and maintaining the integrity of chromosome ends^15^. Based on the results from cellular and animal models, RTEL1 performs two distinct functions essential for telomere maintenance: it facilitates T-loop disassembly and telomeric G4 DNA unwinding. Likewise, RTEL1 is important to cope with replicative stress and to repair DNA interstrand crosslinks^15^.

A number of GWASs and candidate gene studies have identified *RTEL1* variants involved with genetic predispositions to cancers development. A principal component-adjusted GWAS study, comprising over 275 000 autosomal variants among 692 adult glioma cases and 3992 controls, identified two SNPs within intron 12 (rs6010620) and intron 17 (rs4809324) of *RTEL1* that were significantly associated with glioma and astrocytoma predisposition^16^. Similarly, two further glioma GWAS studies, which genotyped over 500 000 tagged SNPs in a total of 1878 cases and 3670 healthy controls, revealed a significant association with SNP (rs6010620) in intron 12 of the *RTEL1* gene^17^. More recently, large-scale association analysis identifies sentinel variants associated with lung adenocarcinoma are located near genes related to telomere regulation including *RTEL1* rs41309931^18^. In addition, *RTEL1* rs2738780, rs7261546 and rs6062299 were found to be associated with lung cancer development in Han Chinese populations in case-control studies^19^.

The emergence of* RTEL1* variants associated with predisposition to cancers has confirmed the hypothesis that genetic factors underlying telomere length have an especially strong influence on cancer development. Definitive proof of such a role has emerged from the existence of genetic variants influencing both telomere length and cancer susceptibility. Nine common genetic variants have been identified that are associated with leukocyte telomere length at a level of genomewide significance^6^, ^20^. Recent studies have evaluated the relationship between these genetic proxies of telomere length and risk of cancer and found evidence suggesting genetically inferred telomere length is associated with increased cancer risk^21^, ^22^. In the last couple of years the approach of using SNPs related to telomere shortening as an instrumental mean to infer the effect of telomeres on cancer etiology has been successfully used in different tumor types such as renal cell carcinoma^23^, adult glioma^24^, breast cancer^25^ and pancreatic cancer^22^. Consistent with this notion, telomere length was found significantly shorter in individuals carrying the *RTEL11* rs2297441 and rs3208008 risk alleles in the current study.

Even though *RTEL1* is known to regulate telomere length, the precise function of *RTEL1* variant genotypes in PCa development remains elusive. Our findings do not imply that telomere length acts directly on cancer risk and could reflect pleiotropic effects of telomere-length loci. It remains to be answered why another SNP *PARP1* rs1136410 cause telomere shortening has no association with PCa risk. One potential explanation might be *RTEL1* function as a tumour-suppressor gene^15^. Definitive proof of such a role has emerged from analysis of mice defective for the RTEL1-PCNA interaction. Loss of the RTEL1-PCNA interaction significantly accelerates the onset of tumourigenesis in mice also deficient for p53^26^. Furthermore, unlike p53 null mice, additional loss of the RTEL1-PCNA interaction results in predisposition to medulloblastomas. These data suggest that tumourigenesis was associated with changes in telomeric homeostasis upon loss of RTEL1-PCNA interaction. With this notion, a more in-depth comparative examination of RTEL1 mRNA transcripts is required.

A large number of intrinsic and extrinsic factors, such as heredity, epigenetics, aging, stress, immune components, and hormones are all states have been previously associated with telomere shortening^2^, ^27^. As such, limitations of the study are mainly related to the scarcity of the relevant exposure data and nature of retrospective study design. Ideally, prostate tissue telomere length would be available as instruments in our current analysis, but leukocytes telomere length would perhaps be the best surrogate to assess the relationship between telomere length and genetic variants. Because there is an intra-individual synchrony in telomere length across the somatic tissues of humans as evidenced by the strong correlations between the telomere lengths in all tissue types^28^. Moreover, the rates of telomere shortening are similar in the somatic tissues^28^. However, future work aims to directly describe the relationships between prostate tissue, leukocytes telomere lengths and PCa cases. Strengths of the study include a significant number of patients, a candidate gene approach, the high plausibility of the association based on the biologic function of selected candidate genes and an impact on telomere length associated with positive markers.

In conclusion, here we present two novel candidates for PCa risk (*RTEL11* rs2297441 and rs3208008). Our findings also suggest that candidate genes encoding proteins with known function in telomere biology variants contribute to telomere length associations. We expect that further replication and mechanistic studies will continue to provide insights into the etiology of PCa.

## Figures and Tables

**Table 1:** Characteristics of PCa Cases and Controls

Variables	Cases (%) N = 1015	Controls (%) N = 1052	*P*
**Age**	69.1 ± 8.2	68.6 ± 8.9	0.828
≤ 64	291 (28.7)	308 (29.3)	
65-75	496 (48.9)	500 (47.5)	
> 75	228 (22.5)	244 (23.2)	
**Smoking status**			
Never	406 (40.0)	412 (39.2)	0.697
Ever	609 (60.0)	640 (60.8)	
**PSA value (ng/ml)**			
< 10	180 (19.4)		
10-20	195 (21.0)		
> 20	552 (59.6)		
Missing	88 (8.7)		
**Gleason score**			
≤ 7 (3+4)	317 (31.2)		
≥ 7 (4+3)	606 (59.7)		
Missing	92 (9.1)		
**Stage of disease**			
I	5 (0.5)		
II	434 (42.8)		
III	142 (14.0)		
IV	356 (35.1)		
Missing	78 (7.7)		

PSA: prostate-specifc antigen

**Table 2:** Associations of Six SNPs with PCa Risk

SNP	Cases (%)	Controls (%)	*P*	OR (95% CI)	*P*^a^
***POT1* rs727505**					
GG	530 (52.2)	543 (51.6)	0.671	1.00	
AG	409 (40.3)	419 (39.8)		1.01 (0.84-1.21)	0.928
AA	76 (7.5)	90 (8.6)		0.86 (0.62-1.20)	0.384
AG/AA vs. GG			0.785	0.98 (0.83-1.17)	0.844
GG/AG vs. AA			0.372	0.86 (0.63-1.18)	0.357
***POT1* rs727506**					
GG	262 (25.8)	286 (27.2)	0.473	1.00	
AG	495 (48.8)	522 (49.6)		1.04 (0.84-1.28)	0.736
AA	258 (25.4)	244 (23.2)		1.14 (0.90-1.46)	0.289
AG/AA vs. GG			0.479	1.07 (0.88-1.30)	0.501
GG/AG vs. AA			0.238	1.11 (0.91-1.36)	0.293
***RTEL1* rs2297441**					
GG	433 (42.7)	505 (48.0)	0.019	1.00	
AG	455 (44.8)	446 (42.4)		1.18 (0.98-1.412)	0.080
AA	127 (12.5)	101 (9.6)		1.44 (1.08-1.93)	0.014
AG/AA vs. GG			0.015	1.23 (1.03-1.46)	0.021
GG/AG vs. AA			0.035	1.33 (1.01-1.76)	0.044
***RTEL1* rs3208008**					
AA	419 (41.3)	490 (46.6)	0.034	1.00	
AC	468 (46.1)	454 (43.2)		1.20 (1.00-1.44)	0.055
CC	128 (12.6)	108 (10.3)		1.37 (1.03-1.83)	0.033
AC/CC vs. AA			0.015	1.23 (1.03-1.46)	0.020
AA/AC vs. CC			0.094	1.25 (0.95-1.64)	0.110
***PARP1* rs1136410**					
AA	383 (37.7)	374 (35.6)	0.463	1.00	
AG	473 (46.6)	496 (47.1)		0.93(0.77-1.12)	0.438
GG	159 (15.7)	182 (17.3)		0.85 (0.66-1.10)	0.215
AG/GG vs. AA			0.303	0.91 (0.76-1.09)	0.284
AA/AG vs. GG			0.317	0.887 (0.70-1.12)	0.314
***PARP1* rs1805414**					
GG	623 (61.4)	663 (63.0)	0.446	1.00	
AG	335 (33.0)	342 (32.5)		1.05 (0.87-1.26)	0.636
AA	57 (5.6)	47 (4.5)		1.29 (0.86-1.93)	0.215
AG/AA vs. GG			0.441	1.08 (0.90-1.29)	0.423
GG/AG vs. AA			0.233	1.27 (0.85-1.89)	0.238

^a^Adjusted for age and smoking status. The results were in bold if *P* < 0.05.

**Table 3:** Associations between Six SNPs and Telomere Length

SNP	Genotype	Short TL, n (%)	Long TL, n (%)	OR (95% CI)^a^	*P*
*POT1* rs727505	GG	109 (52.4)	126 (57.8)	1.00	
	AG	89(42.8)	80 (36.7)	0.78 (0.52-1.16)	0.213
	AA	10 (4.8)	12 (5.5)	1.04 (0.43-2.50)	0.933
*POT1* rs727506	GG	51 (24.5)	58 (26.6)	1.00	
	AG	93 (44.7)	102 (46.8)	0.96 (0.60-1.54)	0.880
	AA	64 (30.8)	58 (26.6)	0.80 (0.48-1.34)	0.390
*RTEL1* rs2297441	GG	85 (40.9)	113 (51.8)	1.00	
	AG	99 (47.6)	78(35.8)	0.85 (0.46-1.57)	0.596
	AA	24 (11.5)	27 (12.4)	0.59 (0.39-0.89)	0.012
*RTEL1* rs3208008	AA	83 (39.9)	112 (51.4)	1.00	
	AC	102 (49.0)	80 (36.7)	0.75 (0.45-0.97)	0.022
	CC	23 (11.1)	26 (11.9)	0.84 (0.45-1.57)	0.581
*PARP1* rs1136410	AA	89 (42.8)	73 (33.5)	1.00	
	AG	94 (45.2)	118 (54.1)	1.53 (1.01-2.31)	0.043
	GG	25 (12.0)	27 (12.4)	1.32 (0.70-2.46)	0.389
*PARP1* rs1805414	GG	117 (56.3)	142 (65.1)	1.00	
	AG	75 (36.1)	67 (30.7)	0.74 (0.49-1.11)	0.143
	AA	16 (7.7)	9 (4.1)	0.46 (0.20-1.09)	0.077

TL: telomere length; ^a^Adjusted for age and smoking status.

## Abbreviations

PCa: prostate cancer
PSA: prostate-specific antigen
SNP: single nucleotide polymorphism
